# Isolation, characterization, proteome, miRNAome, and the embryotrophic effects of chicken egg yolk nanovesicles (vitellovesicles)

**DOI:** 10.1038/s41598-023-31012-0

**Published:** 2023-03-14

**Authors:** Islam M. Saadeldin, Bereket Molla Tanga, Seonggyu Bang, Chaerim Seo, Abdulkadir Y. Maigoro, Heejae Kang, Dabin Cha, Sung Ho Yun, Seung Il Kim, Sanghoon Lee, Jongki Cho

**Affiliations:** 1 Laboratory of Theriogenology, College of Veterinary Medicine, Chungnam National University, 99, Daehak-Ro, Daejeon, 34134 Republic of Korea; 2 Research Institute of Veterinary Medicine, Chungnam National University, Daejeon, 34134 Republic of Korea; 3 Department of Microbiology and Molecular Biology, College of Bioscience and Biotechnology, Chungnam National University, Daejeon, 34134 Republic of Korea; 4 Korea Basic Science Institute (KBSI), Ochang, 28119 Republic of Korea

**Keywords:** Cell proliferation, Embryology

## Abstract

Egg yolk constitutes about a third of the structure of the chicken egg however, the molecular structure and physiological effects of egg yolk-derived lipid membranous vesicles are not clearly understood. In this study, for the first record, the egg yolk nanovesicles (vitellovesicles, VVs) were isolated, characterized, and used as a supplement for porcine embryo culture. Yolks of ten freshly oviposited eggs were filtered and ultracentrifuged at 100,000 × g for 3 h to obtain a pellet. Cryogenic transmission electron microscopy and nanoparticle tracking analysis of the pellet revealed bilipid membranous vesicles. Protein contents of the pellet were analyzed using tandem mass spectrometry and the miRNA content was also profiled through BGISEQ-500 sequencer. VVs were supplemented with the in vitro culture medium of day-7 hatched parthenogenetic blastocysts. After 2 days of blastocyst culture, the embryonic cell count was increased in VVs supplemented embryos in comparison to the non-supplemented embryos. TUNEL assay showed that apoptotic cells were increased in control groups when compared with the VVs supplemented group. Reduced glutathione was increased by 2.5 folds in the VVs supplemented group while reactive oxygen species were increased by 5.3 folds in control groups. Quantitative PCR analysis showed that VVs significantly increased the expression of lipid metabolism-associated genes (monoglyceride lipase and lipase E), anti-apoptotic gene (BCL2), and superoxide dismutase, while significantly reducing apoptotic gene (BAX). Culturing embryos on Matrigel basement membrane matrix indicated that VVs significantly enhanced embryo attachment and embryonic stem cell outgrowths compared to the non-supplemented group. This considers the first report to characterize the molecular bioactive cargo contents of egg yolk nanovesicles to show their embryotrophic effect on mammalian embryos. This effect might be attributed to the protein and miRNA cargo contents of VVs. VVs can be used for the formulation of in vitro culture medium for mammalian embryos including humans.

## Introduction

Egg yolk constitutes approximately one-third of the structure of the chicken egg and is enriched with essential metabolites and nutrients for chicken embryonic development^1^. The composition of egg yolk proteins and RNAs has been explored by several reports^2–5^. Egg yolk is enriched in proteins, cholesterol, and phospholipids and all of them are embedded inside the high-density and low-density lipoprotein micelles or granular particles^6^. Although many studies have provided a theoretical basis, research on ultramicroscopic and compositional compartments of yolk vesicles have not been performed so far.

Extracellular vesicles (EVs) including exosomes and microvesicles have been successfully identified and characterized in most of the biological fluids of humans, animals, and plants including plasma, milk, and saliva^7–10^. However, no work has been published on the isolation and characterization of egg yolk vesicles, to the best of our knowledge. Conversely, egg yolk lecithin was used to manufacture nanosized liposome vesicles for therapeutic use and drug delivery^11–13^.

The embryonic development of chicken embryos was used as a common paradigm to explore the roles of egg components on the early stages of embryonic development. Avian embryos undergo rapid cleavages during the first days of development, and this raises questions about the involvement of egg yolk components in enhancing embryonic development^14^. Pigs are considered a great tool for biomaterial production, organ xenotransplantation, and the development of biomedical models^15^. However, several challenges are facing to establish embryonic stem cells due to the low developmental competence of the in vitro–produced porcine embryos^16,17^. Pig embryonic stem cell research and the implantation process are important links to exploration and action^18–23^.

In the current work, we introduce the term “vitellovesicles” to describe chicken egg yolk lipid nanovesicles and outline ultramicroscopic features and contents of cargo protein and miRNA. Moreover, we studied the potential embryotrophic effects of these egg yolk vesicles on blastocyst culture, apoptosis, and oxidative stress in the porcine model.

## Materials and methods

### Chemicals and reagents

Chemicals and reagents were purchased from Sigma-Aldrich (St. Louis, MO, USA) unless otherwise specified.

### Isolation and characterization of egg yolk vesicles

Egg yolk of freshly oviposited commercially purchased eggs (n = 10) were aseptically aspirated and diluted 10 times with phosphate-buffered saline (PBS), and then centrifuged at 16,000 × g for 30 min to remove the big protein particles. The clear supernatants were microfiltered through 0.4 µm and 0.2 µm microfilters. The filtrates were subjected to ultracentrifugation at 100,000 × g for 3 h using Optima XPN-100 Ultracentrifuge (Beckman Coulter, Indianapolis, IN, USA)^24^. VVs pellets were eluted in 50 µL of PBS solution. VVs were characterized through nanoparticle tracking analysis (NTA, NanoSight NS300, Malvern Instruments, Worcestershire, UK)^25^. 100-nm polystyrene particles (ThermoFisher Scientific, Waltham, MA, USA) were used for the device calibration. 50 µL of eluted VVs was diluted 20 × in 1 × PBS and was loaded into the NTA device. The machine was adjusted to read the mean particle size (diameter), particle concentration, and outlier removal. The reads were set to eleven different positions and two reading cycles per position in each sample. VVs were also visualized through transmission electron microscopy (TEM) and cryogenic TEM (Cryo-TEM)^26,27^. For TEM, 5 μL of eluted VVs was negatively stained with 2% uranyl acetate, moved onto 200-mesh copper grids, dried, and examined through an OMEGA-energy filtering TEM (ZEISS LEO 912, Carl Zeiss, Jena, Germany) at 120 kV. For Cryo-TEM, 3.5 µL of the eluted VVs was applied to glow-discharged Quantifoil R1.2/1.3 Cu 300 grids (Quantifoil, Jena, Germany) and were flash frozen in liquid ethane using Vitrobot mark IV (Thermo Fisher Scientific) set at 100% humidity and 4 °C for the preparation chamber and 5 s of blot time. Cryo-TEM micrographs were imaged on a Glacios microscope (Thermo Fisher Scientific) operated at an accelerating voltage of 200 kV with a 70-µm C2 aperture at an indicated magnification of 73 k × . A Falcon III direct electron detector (Thermo Fisher Scientific) in linear mode was used to acquire images of the samples with a 100-µm objective aperture. The EVs cargo contents of proteins were analyzed through proteomics as discussed below.

### In vitro generation of porcine parthenogenetic embryos

Electric parthenogenetic activation of in vitro matured porcine oocytes was used to generate porcine embryos as showed in previous reports^16,17,28^. Briefly, ovaries were collected from a local slaughterhouse in Daejeon city, and cumulus-oocyte complexes (COCs) were aspirated. Good quality oocytes (homogenous ooplasm and surrounded by compact layers of cumulus cells) were microscopically selected and washed three times in N-2-hydroxyethylpiperazine-N′-ethane-sulphonic acid (HEPES) buffered Tyrode’s medium comprising of 0.05% polyvinyl alcohol (TLH-PVA). COCs were in vitro matured in 500 mL of a maturation medium in 4-well dishes (Nunc, ThermoFisher Scientific, Roskilde, Denmark). Maturation medium was composed of TCM-199 (Gibco, Waltham, MA, USA) supplemented with 10% (v/v) porcine follicular fluid, 0.6 mM cysteine, 0.91 mM sodium pyruvate, 10 ng/mL epidermal growth factor, 1 μg/mL insulin, 10 IU/mL hormones (human chorionic gonadotrophin and equine chorionic gonadotrophin (Daesung Microbiological Labs, Gyeonggi-do, Korea), and 75 μg/mL kanamycin. COCs were matured for 22 h in this medium and then were transferred to the same culture conditions without the presence of hormones for an additional 22 h. Mature COCs were subjected to hyaluronidase (0.6%), gently pipetted to remove cumulus cells and then washed in TLH-PVA and electrically activated inside a glass chamber of BTX Electro-Cell Manipulator 2001 (BTX Inc., San Diego, CA, USA) connected with two electrodes to transmit a single direct current pulse of 1.5 kV/cm for 60 μs. Activation medium comprised mannitol (0.28 M), calcium chloride (0.1 mM), HEPES (0.5 mM), and magnesium sulfate (0.1 mM). Parthenogenetically activated oocytes were cultured in 25 μL of porcine zygote medium-5 (IFP Co. Ltd., Yamagata, Japan) covered with mineral oil in a humidified atmosphere at 38.5 °C (5% O_2_, 5% CO_2_, and 90% N_2_) for 7 days. Blastocysts were collected and zona pellucida was removed after incubation with 0.1% pronase (w/v in PBS) for 30 s and zona-free blastocysts were used for the experiments.

### Embryo attachment and embryonic stem cells outgrowth

Feeder-free culture condition for embryos attachment was used according to a previous report ^29^. The 4-well dishes (Nunc) were cooled and forty μL of microdrops of Matrigel® (BD Biosciences, San Jose, CA, USA) were placed in each well and then incubated for 30 min at 37 °C. Excess Matrigel was removed and 40 μL of culture medium were placed over the drop and covered with mineral oil. Culture medium comprised Dulbecco’s modified eagle medium/nutrient mixture F-12, fetal bovine serum (10% v/v), 0.1 mM β-mercaptoethanol, 1% nonessential amino acids (Invitrogen Corporation, Carlsbad, CA, USA), and 1% penicillin/streptomycin (100 U/mL penicillin and 100 µg/mL streptomycin). Zona-free blastocysts were washed with the culture medium before placed in the microdrops and cultured in a humidified atmosphere of 5% CO_2_ at 38.5 °C. On days 2–5 of culture, embryos were checked for attachment and outgrowths. VVs (1.6 × 10^8^/ml) were supplemented with the in vitro culture medium (n = 160, 8 replicates of 10 blastocysts from each control and VVs-supplemented group), while control group was not supplemented with VVs.

### TdT-mediated deoxyuridine triphosphate (dUTP-X) nick end labeling (TUNEL) assay

Detection of apoptosis was performed after labeling of DNA strand breaks by using in situ–cell death detection TUNEL assay Kit (Roche Holding AG, Basel, Switzerland). Embryos at the designed days were fixed in 4% paraformaldehyde, permeabilized in 0.1% TritonX, washed in PBS, and then placed in the enzyme (TdT) and a label (fluorescein-dUTP) solutions for 1 h at 37 °C. Vectashield antifade mounting medium was used for nuclear staining. Green fluorescence signals were captured, counted, and calculated using ImageJ software.

### Measuring reduced glutathione and reactive oxygen species in embryos

Intracellular levels of reactive oxygen species (ROS) were measured after staining with 2′,7′-dichlorodihydrofluorescein diacetate (H2DCFDA; Invitrogen Corporation, Carlsbad, CA, USA). ROS accumulation was indicated as green, fluorescent signals. Intracellular levels of reduced glutathione (GSH) were measured after staining with CellTracker™ Blue including a blue fluorescent dye 4-chloromethyl-6,8-difluoro-7-hydroxycoumarin (CMF2HC). In each experiment, twenty-five embryos (n = 5 for 5 replicates) from each treatment group were used. Fluorescence signals were visualized through an epifluorescence microscope (Leica DM IRB; Leica Microsystems, Wetzlar, Germany) associated with ultraviolet filters (460 nm for ROS and 370 nm for GSH). Fluorescent images were processed through ImageJ (version 1.41; National Institutes of Health, Bethesda, MD, USA, https://imagej.nih.gov/ij/) to measure the fluorescence intensity after normalization to corresponding control group.

### Relative quantitative polymerase chain reaction (RT-qPCR)

RNeasy Micro Kit (Qiagen GmbH, Hilden, Germany) was used to extract total RNA from the embryos (n = 5, 5 replicates). The concentration and quality of RNA was determined with NanoDrop 2000 (Thermo Scientific, Waltham, MA, USA). Reverse transcription was performed through using 2X RT Pre-Mix of QuantiNova Reverse Transcription Kit (Qiagen). Real time polymerase chain reaction (qPCR) was conducted by the CFX Connect Real-Time PCR system (Bio-Rad) and SYBR 2X Real-Time PCR Pre-Mix (BioFACT, Daejeon, Korea). Details about the primers, examined genes, and amplicon size are illustrated in Table 1. Quantification of the target transcripts were compared to those of housekeeping genes (ACTB-mRNA and U6-snRNA) through the ΔΔCt method.

**Table 1:** Primers used for RT-qPCR.

Name	Sequence 5′ → 3′		Product size	Accession No
	Forward	Reverse		
U6	GCTTCGGCAGCACATATACTAAAAT	CGCTTCACGAATTTGCGTGTCAT	89	NR_004394.1
Bax	ACTTCCTTCGAGATCGGC	GGCCACGAAGATGGTCAC	110	XM_013998624.2
Bcl2	TTCTCTCGTCGCTACCGC	CCAGTTCACCCCATCCCT	123	NM_214285.1
ACTB	CCCTGGAGAAGAGCTACGAG	GGTGCCACCCGACAGCAC	206	XM_021086047.1
SOD1	ACCTGGGCAATGTGACTG	CATTTCCACCTCTGCCCA	147	NM_001190422
MGLL	TATGAGGGTGCCTACCAC	GTCCTTTGGGAGACCCAT	92	NM_001143718.1
LIPE	TGACTCAGACCAGAAGGC	CAGCTCCAGGAAGGAGTT	115	NM_214315.3

*U6* RNU6-1 RNA, U6 small nuclear 1 (*house-keeping snRNA* https://www.ncbi.nlm.nih.gov/gene/26827), *Bax* BCL2 associated X, apoptosis regulator (*causes apoptosis* https://www.ncbi.nlm.nih.gov/gene/396633), *Bcl2* BCL2 apoptosis regulator (*antiapoptotic* https://www.ncbi.nlm.nih.gov/gene/100049703), *SOD1* superoxide dismutase 1, *MGLL* Monoglyceride lipase, *LIPE* Lipase E, hormone-sensitive type. Cycling conditions of the PCR were 95 °C (1 min) for initial denaturation followed by forty cycles of 95 °C (5 s), 60 °C (30 s), and 72 °C (30 s). The specificities of primers were determined using the melting curve protocol from 65 to 95 °C and was confirmed with single peaks in the melt curves, gel electrophoresis, and DNA-free samples.

### Preparation of protein fraction of VVs and proteomic analysis

Protein isolation and proteomics were conducted according to Lee et al.^26^. Eluted VVs protein contents were measured through the bicinchoninic acid method. Sodium dodecyl sulfate–polyacrylamide gel electrophoresis (SDS-PAGE) was used for protein fractionation. Gels were stained in NH_4_CO_3_ (10 mM) and acetonitrile (50%) for Coomassie Brilliant Blue staining^30^, then rinsed twice with distilled water and acetonitrile (100%). Gels were then dried in a speed vacuum evaporator and then treated with NH_4_HCO_3_ (100 mM) and dithiothreitol (10 mM) at 56 °C, before treated with 100 nM iodoacetamide to reduce alkylate S–S bridges. Gels were then washed in distilled water and then dried in a speed vacuum evaporator. Gels were kept for 12–16 h in trypsin (10 ng/mL) and NH_4_HCO_3_ (50 mM) at 37 °C for protein digestion. The resulting peptides were retrieved after treatment and suspension in 0.5% trifluoroacetic acid, purified, and concentrated through MGU30-C18 trapping columns (LC Packings). MS and MS/MS spectra were captured through LTQ-Velos ESI ion trap mass spectrometer (Thermo Scientific, Waltham, MA). The data-dependent mode was used for fragmentation of ten most abundant peaks from the full MS scan with 27% normalized collision energy. The eluted peptides were made to enter into the mass spectrometer at an electrospray voltage of 2.3 kV. The maximum ion injection times used were 100 ms for the MS scan and 100 ms for the MS/MS scans. The automatic gain control target settings were 5.0 × 10^4^ for the MS scan mode and 1.0 × 10^5^ for the MS/MS scan mode. The dynamic exclusion duration was set at 180 s and exclusion mass width 0.5 Da. The mass range for acquiring MS spectra was 150–2000 m/z. Protein identification were performed by MASCOT 2.4 with the false discovery rate at 1%, as a cutoff value. Relative quantitation of proteins was determined through the exponentially modified protein abundance index (emPAI) and were expressed as mol %. Protein functions and gene ontology (GO) were analyzed a web-based tool provided by DAVID (NIAID/NIH; https://david.ncifcrf.gov/)^31,32^. GO functional analysis was performed using the egg yolk vesicles proteomic data, 448 proteins accessions were process for functional analysis. DAVID database (https://david.ncifcrf.gov/) was used to convert the accessions into official names, and further used for GO functional annotation of the target genes. This was used for identification of biological process (BP), molecular function (MF), and cellular component (CC) and pathway. Bioinformatics tools were used for the construction of bubble plots (http://www.bioinformatics.com.cn/plot_basic_gopathway_enrichment_bubbleplot_081_en). Protein–protein interaction was performed through using String database. The generated network was further processed using String plugin in Cytoscape using confidence score cutoff (0.4).

### Small RNA sequencing and bioinformatic analysis

#### Transcriptome library construction and sequencing

The total RNA of VVs was isolated from three random samples as mentioned above and integrity and concentration of collected small RNAs (sRNAs) were evaluated through Agilent 2100 Bioanalyzer (Agilent RNA 6000 Nano Kit, Agilent Technologies, Santa Clara, CA, USA). sRNA sequencing libraries were executed following the BGISEQ-500 Small RNA unique molecular identifier (UMI) Library Construction Protocol. Each 1 ng of sRNA sample was adaptor-ligated in consequential steps: connection of the 3´-adaptor system at 70 °C for 2 min and 25 °C for 2 h, then UMI-labeled primers were added at 65 °C for 2 min, ramp to 4 °C at a rate of 0.3 °C/s. Finally, a 5´-adaptor system was added at 70 °C for 2 min, 25 °C for 1 h. After adaptors ligation, the first strand master mix was mixed with SuperScript II (Invitrogen Corporation) for reverse transcription at 42 °C for 1 h followed by enzyme inactivation at 70 °C for 15 min. cDNA was amplified through PCR using primers and PCR master mix in the following cycling conditions: initial 95 °C / 3 min, 20 cycles of 98 °C / 20 s, 56 °C / 15 s, 72 °C / 15 s, and final extension at 72 °C /10 min. PAGE gel electrophoresis was used to purify the final PCR products and then were dissolved in an elution buffer. The resulting PCR product was heat-denatured and circularized by the split oligo sequence to be single-stranded circle DNAs (ssCir DNA) that were considered as the final library. Constructed libraries were analyzed through the Agilent 2100 Bioanalyzer and then were amplified with phi29 to make DNA nanoballs (DNBs) that contain > 300 copies per ball. The DNBs were transferred into a patterned nanoarray and single end 50 bases reads were generated during sequencing by synthesis. BGISEQ-500 sequencer was used for the RNA-Seq libraries and base-calling was conducted by its accompanied software (version #0.3.8.111).

### Sequencing data analysis

RNA-seq libraries were checked for quality through FastQC v0.11.5 software (https://www.bioinformatics.babraham.ac.uk/projects/fastqc/). The raw reads that contain the sequence of the primers/adaptors, high content (10%) of unknown bases (N), and low-quality reads (≤ 5 bases) were identified as ‘dirty’ reads and were excluded to reduce data noise. The remaining “clean” reads were stored in the FASTQ format and mapped to reference *Gallus* genome (NCBI: bGalGal1.mat.broiler.GRCg7b), and small RNA sequences from miRBase (version 22.1, https://www.mirbase.org/) with mirDeep2 software (miRDeep2.0.0.8, https://github.com/Drmirdeep/miRDeep2). Read numbers of known and predicted novel miRNAs and small RNAs of each sample were counted Using miRDeep2.pl script. Moreover, the ortholog miRNAs from human, pig, rat, and mouse were explored and compared with the resultant chicken miRNAs.

### Target genes and GO functional analysis

Using (http://www.mirdb.org/mirdb/ontology.html) database, target genes based on ontology for each ortholog were identified. Target genes with score ≥ 99 were considered for the analysis. Only 25 orthologs were considered for the matured miRNA, and 4 orthologs for Novel mi-RNA. Other miRNA orthologs that have target genes with less score were not considered. DAVID database was further used for GO functional annotation of the target genes. This was used for identification of biological process (BP), molecular function (MF), and cellular component (CC) and pathway. Bioinformatics tools were used for the construction of bubble plots (http://www.bioinformatics.com.cn/plot_basic_gopathway_enrichment_bubbleplot_081_en).

### Statistical analyses

Samples homogeneity and normality of variance distribution were validated through Lieven’s test and Kolmogorov–Smirnov test, respectively. Values were presented as mean ± standard error of means (SEM) and analyzed through an unpaired Student’s t-test or with univariate analysis of variance (ANOVA) followed by Tukey’s multiple comparison test. Statistical analyses were conducted with GraphPad Prism 5 (GraphPad Software Inc., San Diego, CA, USA, https://www.graphpad.com/) and differences were considered significant when *p* < 0.05 or *p* < 0.01. was used for statistical analyses.

## Results

### Isolation, characteristics, and protein cargo contents of VVs

**Figure 1:**
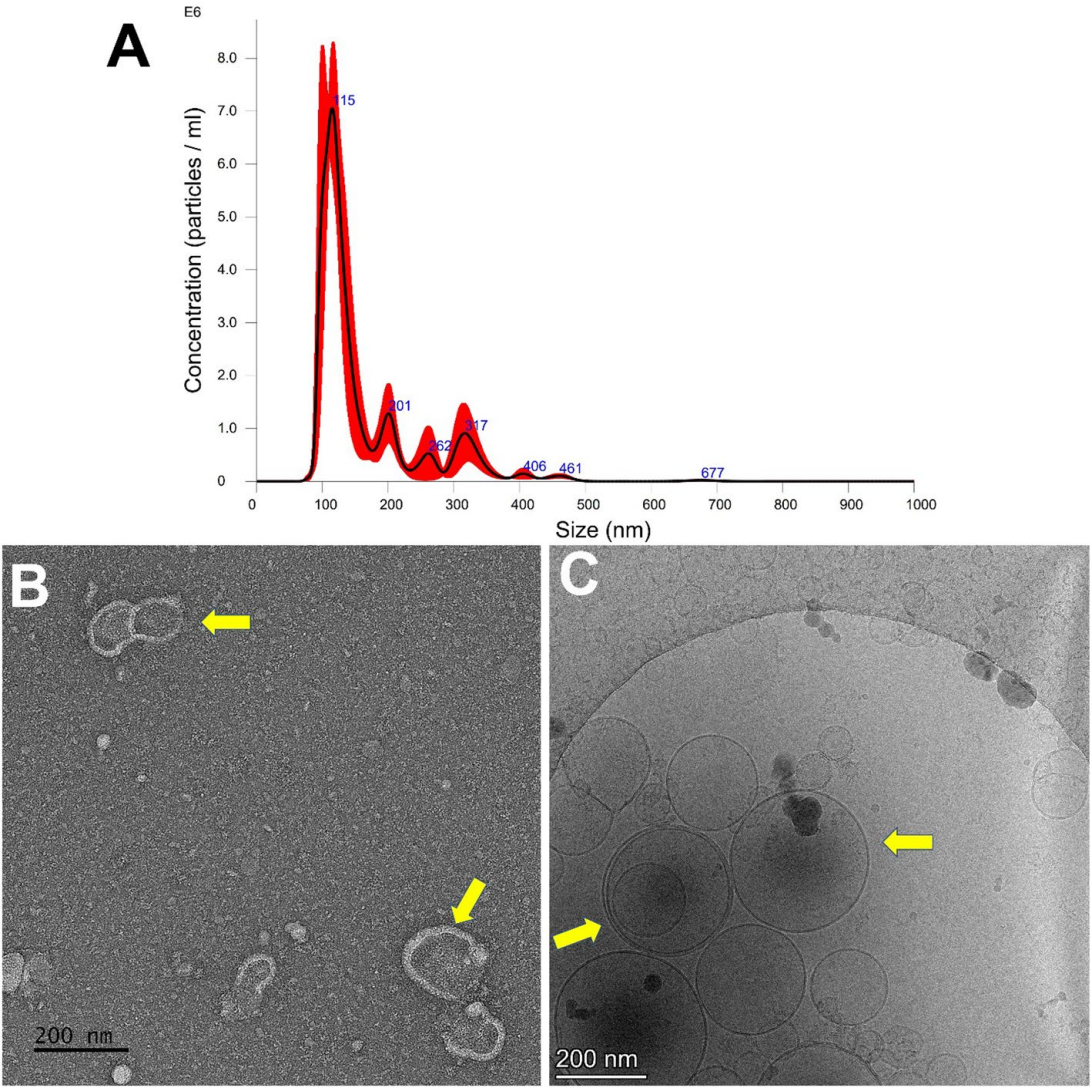
Isolation and characterization of VVs. VVs were isolated by the targeted nanofiltration method and were characterized using NanoSight nanoparticle tracking analysis and showed an average diameter of 158.6 ± 20.5 nm with a concentration of 1.1 × 10^8^ particles/mL (dilution factor is 20 ×) (**A**). VVs were visualized by transmission electron microscopy (**B**), and cryo-transmission electron microscope (**C**) and showed bilipid vesicles (yellow arrows). The scale bar = 200 nm.

**Figure 2:**
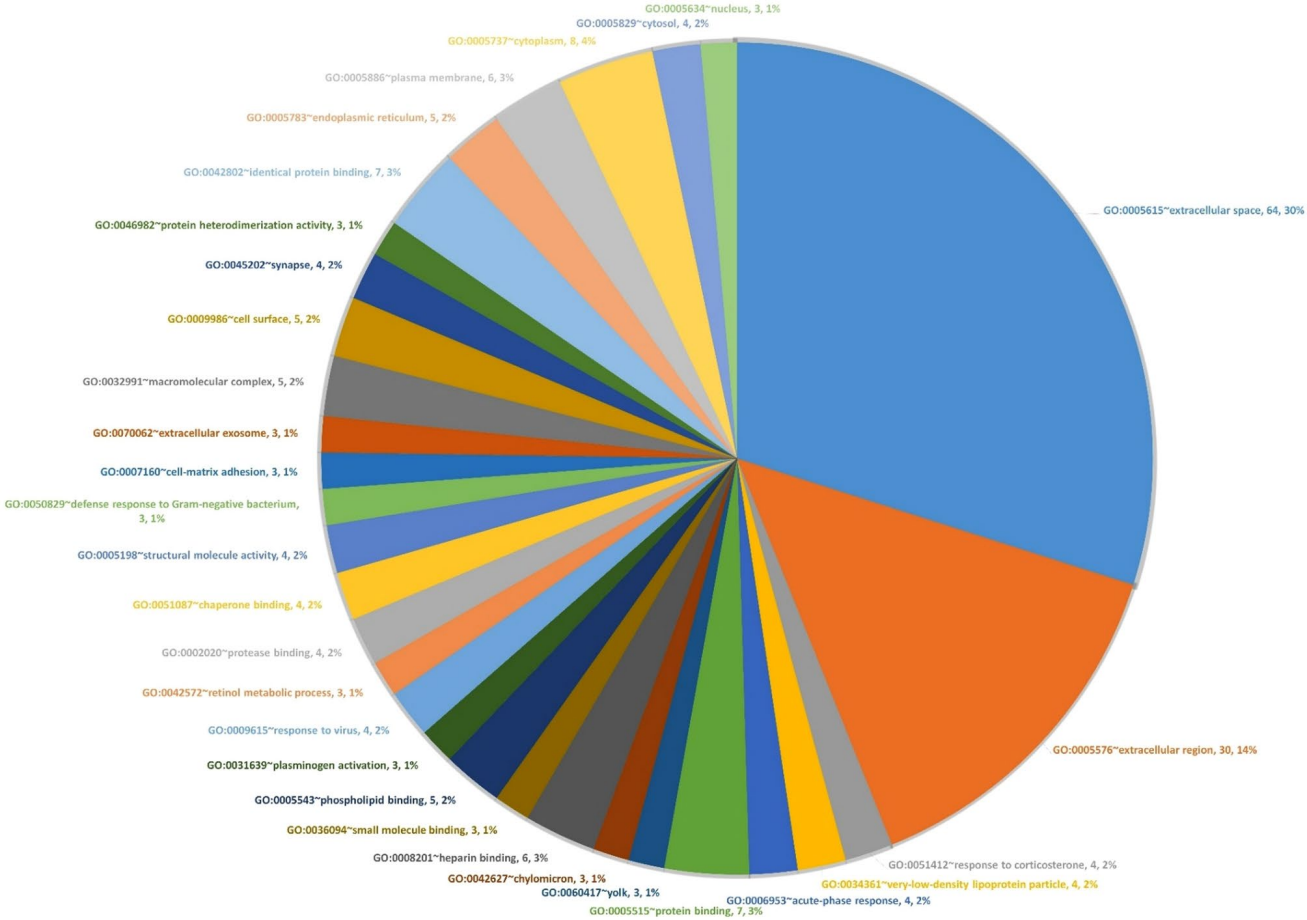
Gene ontology (protein count and percentage) and major functional processes of the proteins in VVs. Bioinformatics analysis showed involvement of VVs protein in different cellular compartments such as extracellular space, plasma membrane, exosomes, and other structural and metabolic processes.

NTA results showed the existence of 1.6 × 10^10^ particles/mL with mean particle size 158.6 ± 20.5 nm in the isolated VVs. Furthermore, Cryo-TEM images indicated the presence of lipid bilayer vesicles (Fig. 1). The protein contents of isolated VVs were analyzed using proteomics and 498 protein reads were identified (Supplementary Table 1), of which, the top twenty proteins accounted around 83.5% of the total proteins of VVs, including proteins associated with different cellular functions such as protein (albumin, vitellogenins, ovalbumin, fibrinogen, angiotensinogen, transthyretin, and insulin-like growth factor binding protein) and lipid metabolism (apovitellenin-1, apolipoprotein A–I, apolipoprotein B, and lipocalin) in addition to the protective immune proteins (lysozyme C, ovoinhibitor, and complementary system) and immunoglobulins (Table 2, and Supplementary Figures S1–S3). Moreover, the entire proteins showed involvement in different cellular components and biological processes such as protein and lipid metabolism and signaling as well as the exosomal structures (Fig. 2). Furthermore, oxidative defense proteins were also expressed in VVs such as thioredoxin domain-containing protein (accession # A0A1D5PLH7). Importantly, proteomics of VVs revealed exosomes-related tetraspanins and pentaspan transmembrane glycoprotein such as CD81 (accession # F1NW06), heat shock protein family A (Hsp70) member 8 (HSPA8; accession # A0A1D5PFJ6), cystatin C (accession #A0A1L1S0C3), annexin A6 (accession #A0A1D5PY67), and prominin or CD133 (accession # A0A3Q2U8I8) (Supplementary Table S1).

**Table 2:** Top 20 proteins detected in VVs.

UniProt accession	Description	Mol% ± SD
P19121	Albumin	33.03 ± 6.2
P02659	Apovitellenin-1	16.69 ± 2.5
P02789	Ovotransferrin	12.01 ± 1.7
P08250	Apolipoprotein A-I	5.33 ± 0.97
A0A3Q2UDA5	Ig-like domain-containing protein	4.74 ± 0.96
F1NV02	Apolipoprotein B	4.43 ± 1.01
P02845	Vitellogenin-2	2.29 ± 0.83
A0A3Q2U347	Vitellogenin-3	0.97 ± 0.97
A0A1D5NUW2	Vitellogenin-1	0.63 ± 0.12
P01012	Ovalbumin	0.56 ± 0.11
R9PXM5	Immunoglobulin lambda-like polypeptide 1	0.50 ± 0.13
F1NUL9	Fibrinogen beta chain	0.40 ± 0.08
P00698	Lysozyme C	0.36 ± 0.1
F1NDH2	Angiotensinogen	0.33 ± 0.08
A0A1D5PEY8	Lipocln cytosolic FA-bd domain-containing protein	0.32 ± 0.07
E1BV78	Fibrinogen C-terminal domain-containing protein	0.29 ± 0.07
E1C7C1	Complement component C8 beta chain	0.22 ± 0.02
P10184	Ovoinhibitor	0.18 ± 0.04
F1NI07	Insulin-like growth factor binding protein acid labile subunit	0.17 ± 0.03
A0A1L1RWR0	Transthyretin	0.15 ± 0.02

### GO functional analysis and protein–protein interaction (PPI)

Using the converted accession IDs, the proteins were utilized for the functional analysis from DAVID. The GO-BP analysis shows that, the genes are highly enriched in negative regulation of peptidase activity (GO:0,010,466), and negative regulation of endopeptidase activity (GO:0,010,951). GO-CC are enriched in extracellular region (GO:0,005,576), and extracellular region part (GO:0,044,421). While GO-MF from our data shows enrichment in endopeptidase and serine type endopeptidase inhibitor activity (GO:0,004,866), and peptidase regulator activity (GO:0,061,134) as shown in Supplementary Figure SF1. Pathway analysis was found to be significantly enriched in phagosome (gga04145) and regulation of actin cytoskeleton (gga04810) among others as shown in (Supplementary Figure SF2). The ClueGo functional analysis from the Cytoscape shows the highly enriched BP, CC, and MF from the PPI network. The enriched BP include blood coagulation, peptidase activity, and homotypic cell–cell adhesion among others. While highly enriched CC include extracellular space, and cell adhesion molecule binding among others. MF include plasma lipoprotein particles, and extracellular exosome as shown in (Supplementary Figure SF1). pathway analysis was performed by integrating the KEGG, reactome and WikiPathway shows phagosome and glycolysis/gluconeogenesis as highly enriched pathways as shown in (Supplementary Figure SF3).

### MicroRNA contents of VVs

**Figure 3:**
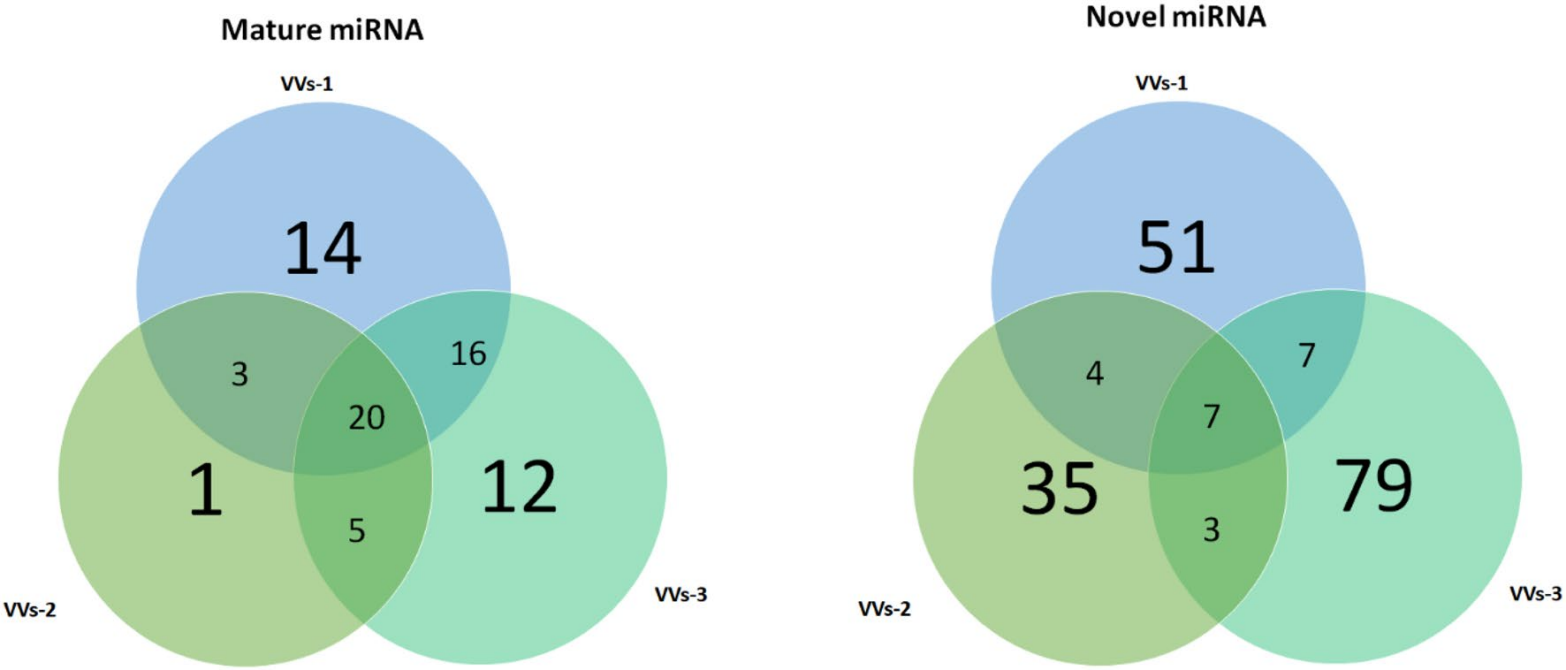
Venn diagram shows the numbers of matched mature and novel miRNAs in three samples of VVs.

The miRNA proportion was around 4% of the whole detected RNAs (Supplementary Figure SF4). Among the 25,653,000 raw tags, we clarified around 14,138,572 reads of average base pair length 19 bp and after exclusion of all reads less than 17 bp, we identified 10,479 mapped miRNAs according to miRBase 22.1 database (Supplemental Tables S2 and S3). Among the identified siRNAs, around 71 mature miRNA and 186 novel mature miRNAs were detected in VVs (Fig. 3). Twenty miRNAs were expressed in all analyzed samples, while still there are some variations in the detected sequences in the analyzed samples. Among these commonly expressed miRNAs, the top 10 miRNAs by reads count were gga-miR-92-3p, gga-miR-21-5p, gga-miR-199-5p, gga-miR-22-5p, gga-miR-7, gga-miR-31-5p, gga-miR-181a-5p, gga-miR-429-5p, gga-miR-1416-5p, and gga-miR-26a-5p, that showed similar ortholog with pigs miRNA library except for the gga-miR-1416-5p that showed no similarity with the pig or with other species. These top 10 miRNA occupy around 66.31 ± 5.2% from the total reads of the mature miRNAs in VVs. Moreover, from these mature miRNAs, 21 miRNAs were found to affect the embryonic and stem cell development in different mechanisms such as embryo implantation and viability as illustrated in Table 3. Interestingly, these miRNAs are of similar ortholog with pigs (*Sus scrofa*) and humans (*Homo sapiens*) according to miRBase 22.1 database. The known miRNAs in *Homo sapiens*, (hsa, 2,656), *Gallus* (gga, 1,235), and *Sus scrofa* (ssc, 457) (Supplementary Tables S4–S6). Interestingly, data indicated the presence of 186 novel miRNA, and of these, seven are matched to the sequenced database of porcine, human, and mouse species. Additionally, 32 novel miRNA were similar to the human miRNA database, three were similar to mouse (*Mus musculus*, mmu) sequences, and two were similar to rat (*Rattus norvegicus*, rno) sequence (Supplementary Table S7).

**Table 3:** Mature and novel chicken (*Gallus*, gga) miRNAs detected in VVs with their ortholog porcine (*Sus scrofa*, scc) miRNA and their functions related to the development of embryos and stem cells.

miRNA	Functions	Referencess	Reads count		
			VVs-1	VVs-2	VVs-3
gga-miR-21-5p ssc-miR-21-5p	Potential biomarkers for establishing pregnancy	^68^	2162	128	2174
gga-miR-122-5p ssc-miR-122-5p	Stimulates DNA synthesis and inhibits apoptosis of human spermatogonia stem cells	^69^	268	120	256
gga-miR-26a-5p ssc-miR-26a	Regulates gene expression involved in embryo development and implantation in porcine endometrial luminal epithelial cells	^70^	432	6	324
gga-miR-7 ssc-miR-7-5p	Related to development of preimplantation embryos by targeting KLF4 mRNA	^71^	1578	264	1401
gga-miR-100-5p ssc-miR-99b	Expressed in the egg yolk	^5^	119	69	37
gga-miR-191-5p ssc-miR-191	A potential biomarker for implantation Upregulated in culture media of implanted human embryo on day-5 of development	^72^ ^73^	5	2	108
gga-miR-184-5p ssc-miR-184	Regulates epidermal differentiation	^74^	1	76	0
gga-miR-202-5p ssc-miR-202-5p	A germ plasm-specific microRNA in zebrafish Determines embryo viability during mid-blastula transition	^75^ ^52^	646	0	766
gga-miR-451 ssc-miR-451	Involves in mouse embryo implantation	^76^	86	0	166
aucacauugccagggauuucc ssc-miR-23a	Regulates gene expression at the maternal-conceptus interface	^77^	1254	0	250
gga-miR-30d ssc-miR-30c-5p	Biomarker for hampered preimplantation embryo developmental competence Expressed in the egg yolk	^78^ ^5^	0	2	63
gga-miR-204 ssc-miR-204	Regulates cortical morphogenesis	^79^	0	3	1204
gga-miR-125b-5p ssc-miR-125b	Prevents apoptosis in mouse embryos	^80^	0	0	422
	Regulates the expression of maternal genes in preimplantation embryo	^81^			
	Promotes early germ layer specification in human embryonic stem cells	^82^			
	Affects trophoblast gene expression and cell functions in early pregnancy	^83^			
	Regulates gene expression involved in embryo development and implantation in porcine endometrial luminal epithelial cells	^70^			
gga-miR-128–2-5p ssc-miR-128	Regulates the proliferation and neurogenesis of neural precursors	^84^	0	0	111
gga-miR-138-5p ssc-miR-138	Regulates embryo implantation and early pregnancy	^85^	0	0	78
gga-miR-145-5p ssc-miR-145-5p	Associated with human embryo implantation defects	^86^	0	0	1
gga-miR-205a ssc-miR-205	Involves in oocyte-to-embryo transition in pigs	^87^	0	0	86
gga-miR-460a-5p ssc-miR-20a-3p	Critical for normal embryogenesis by targeting vsx1 mRNA in fish	^88^	0	0	48
gga-miR-133a-5p ssc-miR-133a-3p	Regulates proliferation and differentiation of embryonic myoblasts	^89^	22	0	0
gga-miR-193a-5p ssc-miR-193a-5p	Associated with high quality embryos	^90^	0	126	0
gga-miR-196-5p ssc-miR-196a	Involved in mammalian limp development and embryogenesis	^91,92^	15	0	

### miRNAs target genes and GO functional analysis

Using the 25 miRNAs from the matured miRNA ortholog. Total of 306 target genes were identified. hsa-miR-30a-5p has the highest number target genes. For the novel miRNA data, 71 target genes were identified from the 4 miRNAs ortholog with hsa-miR-23a-3p has the highest number of target genes. For the matured miRNA data, the GO-BP analysis shows that, the target genes are highly enriched in nervous system development (GO:0,007,399), and positive regulation of cell metabolic process (GO:0,009,893). GO-CC are enriched in cytosol (GO:0,005,829), and nucleoplasm (GO:0,005,654). While GO-MF from our data shows enrichment in sequence-specific double-stranded DNA binding (GO:1,990,837), and double-stranded DNA binding (GO:0,007,399) as shown in Supplementary Figure SF5. On the other hand, the novel miRNA GO-BP was found highly enriched in phosphate-containing compound metabolic process (GO:0,006,796). The GO-CC is enriched in chromatin (GO:0,000,785). While the GO-MF is found to be significantly enriched phosphoric ester hydrolase activity (GO:0,042,578) among others as shown in Supplementary Figure SF6.

### Effect of VVs on embryo attachment and outgrowths

**Figure 4:**
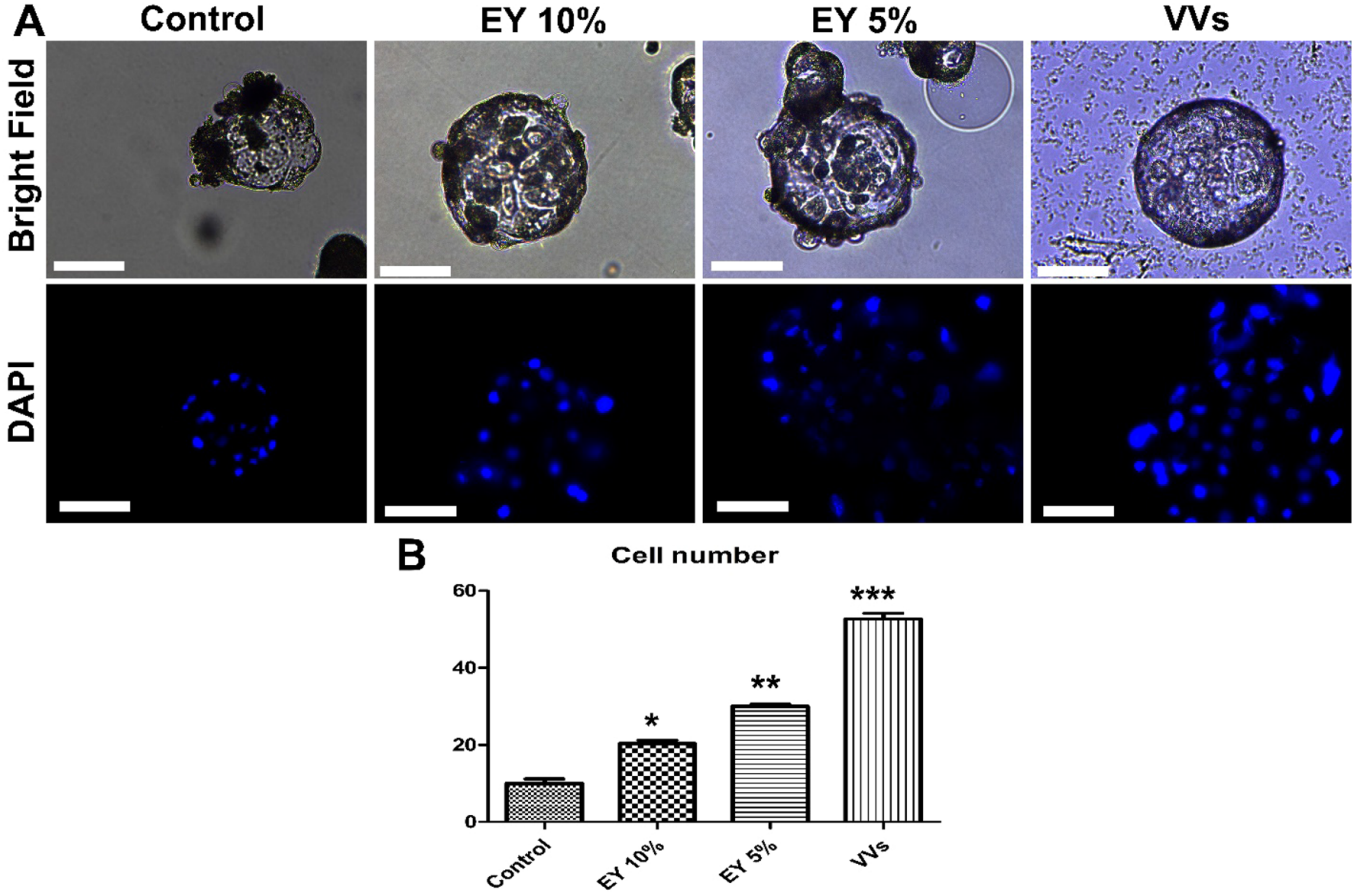
The effects of egg yolk and egg yolk vesicles (VVs) on porcine embryo development. (**A**) Day-7 zona-free embryos (n = 20, 3 replicates) were cultured in micro drops of culture medium in a humidified atmosphere of 5% CO_2_ for 48 h. The control group was cultured in a plain culture medium and the groups were supplemented with egg yolk 10% and 5% and VVs of 2.6 × 10^6^ particles/mL. Scale bar = 100 µm. All groups were imaged in a bright field before staining with 4′,6-diamidino-2-phenylindole stain. (**B**) Graph shows the cell number in the groups, and values were compared using ANOVA followed by Tukey’s test to determine the difference among the groups. Values (mean ± SD) denoted by asterisks (*, **, and ***) were considered statistically different (*p* < 0.05).

**Figure 5:**
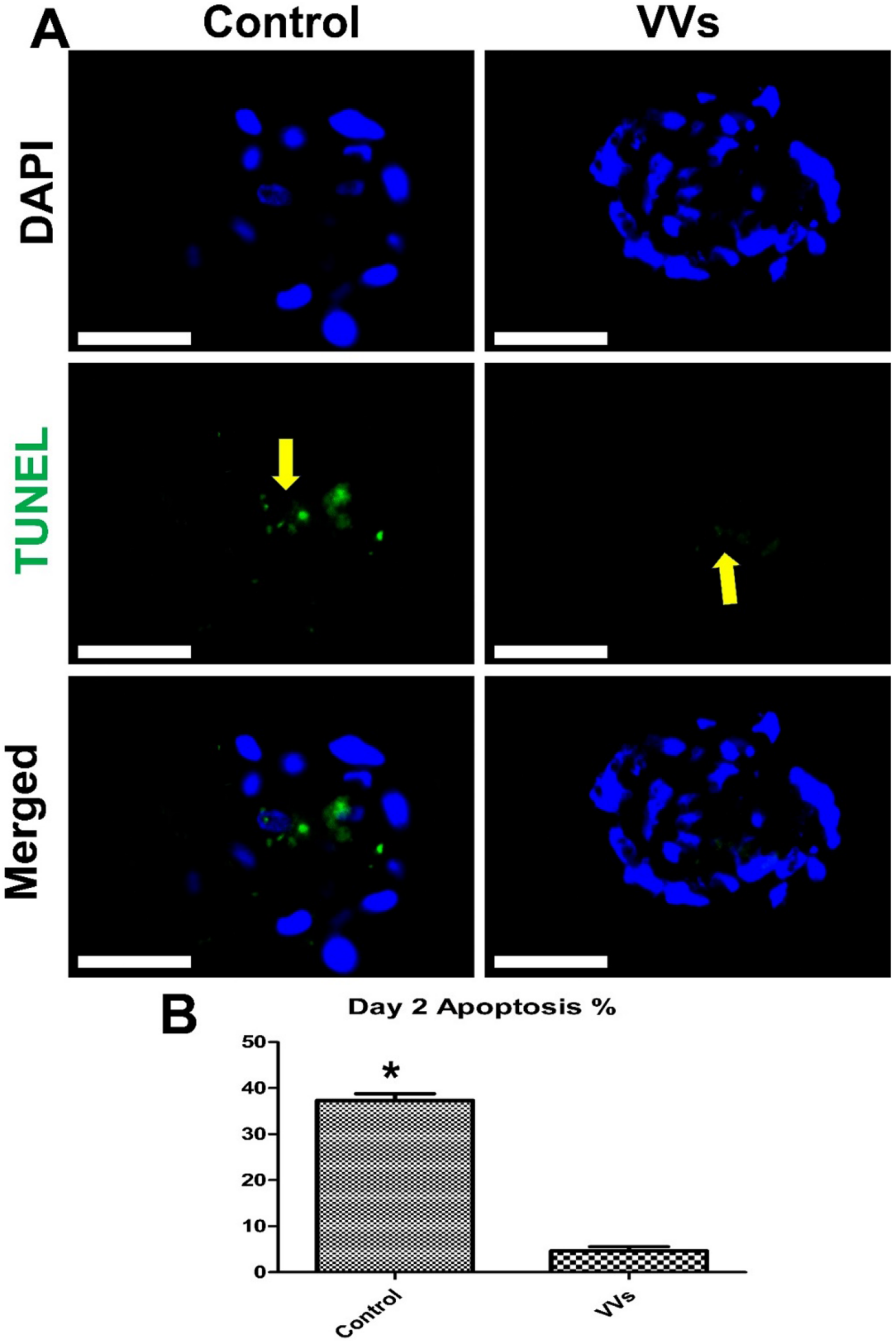
Effects of VVs on porcine embryo apoptosis. (**A**) Day-7 zona-free embryos (n = 20, 3 replicates) were cultured on in micro drops of culture medium in a humidified atmosphere of 5% CO_2_ for 48 h. VVs of 2.6 × 10^6^ particles/mL were supplemented to treatment group and compared with the control non-supplemented group. All groups were examined through TUNEL assay and contrasted with 4′,6-diamidino-2-phenylindole stain. Apoptotic nuclei showed green fluorescence signals (yellow arrows). Scale bar = 100 µm. (**B**) The graph shows the apoptosis percentage in the two groups, and values were compared using Student’s t-test. Value (mean ± SD) denoted by an asterisk (*) was considered statistically different (*p* < 0.05).

**Figure 6:**
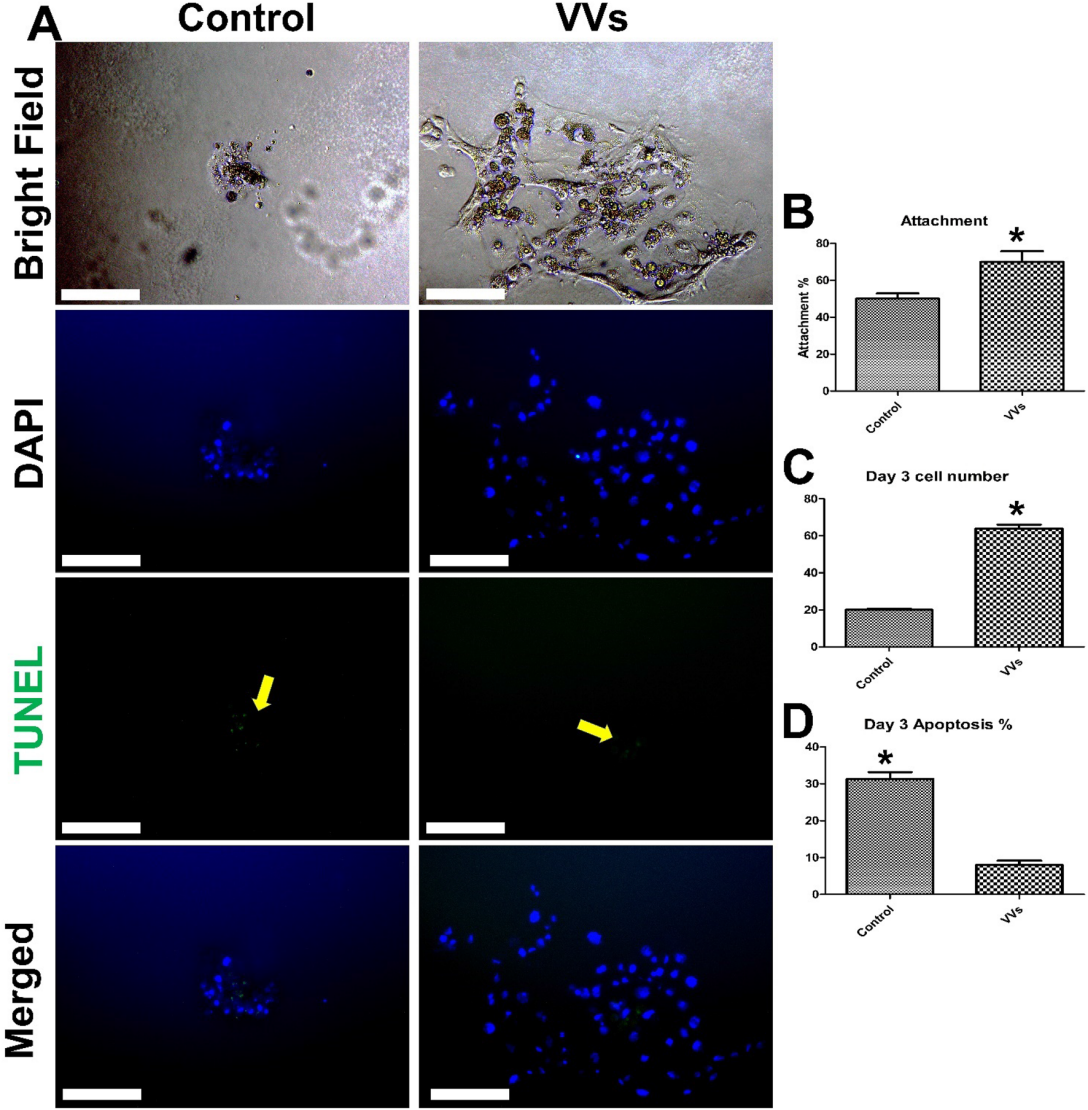
Effects of VVs on porcine embryo attachment and development. (**A**) Day-7 zona-free embryos (n = 20, 3 replicates) were cultured on Matrigel-coated dishes in micro drops of culture medium in a humidified atmosphere of 5% CO_2_ for 72 h. The control group was cultured in a plain culture medium and the VVs groups was supplemented with VVs of 2.6 × 10^6^ particles/mL. All groups were imaged in a bright field before staining with TUNEL assay and contrasted with 4′,6-diamidino-2-phenylindole stain. Scale bar = 100 µm. (**B**, **C**, and **D**) Graphs show the attachment percentage, the cell number in the groups, and the apoptosis percentages, respectively. Values (mean ± SD) were compared using Student’s t-test. Values denoted by an asterisk (*) were considered statistically different (*p* < 0.05).

VVs supplementation for 1.5 day increased embryonic cell number in comparison to the whole egg yolk supplementation of either 10% or 5% and the control non-supplemented group (Fig. 4). Moreover, VVs significantly reduced the apoptosis incidence in day-2 cultured embryos (Fig. 5). Embryo development and outgrowths were daily checked for five subsequent days in VVs supplemented or control groups and VVs increased the embryonic attachment, embryonic cell outgrowths, and cell numbers, and reduced the apoptotic cells (Fig. 6).

### Effect of VVs on oxidative stress and gene expression of embryos

**Figure 7:**
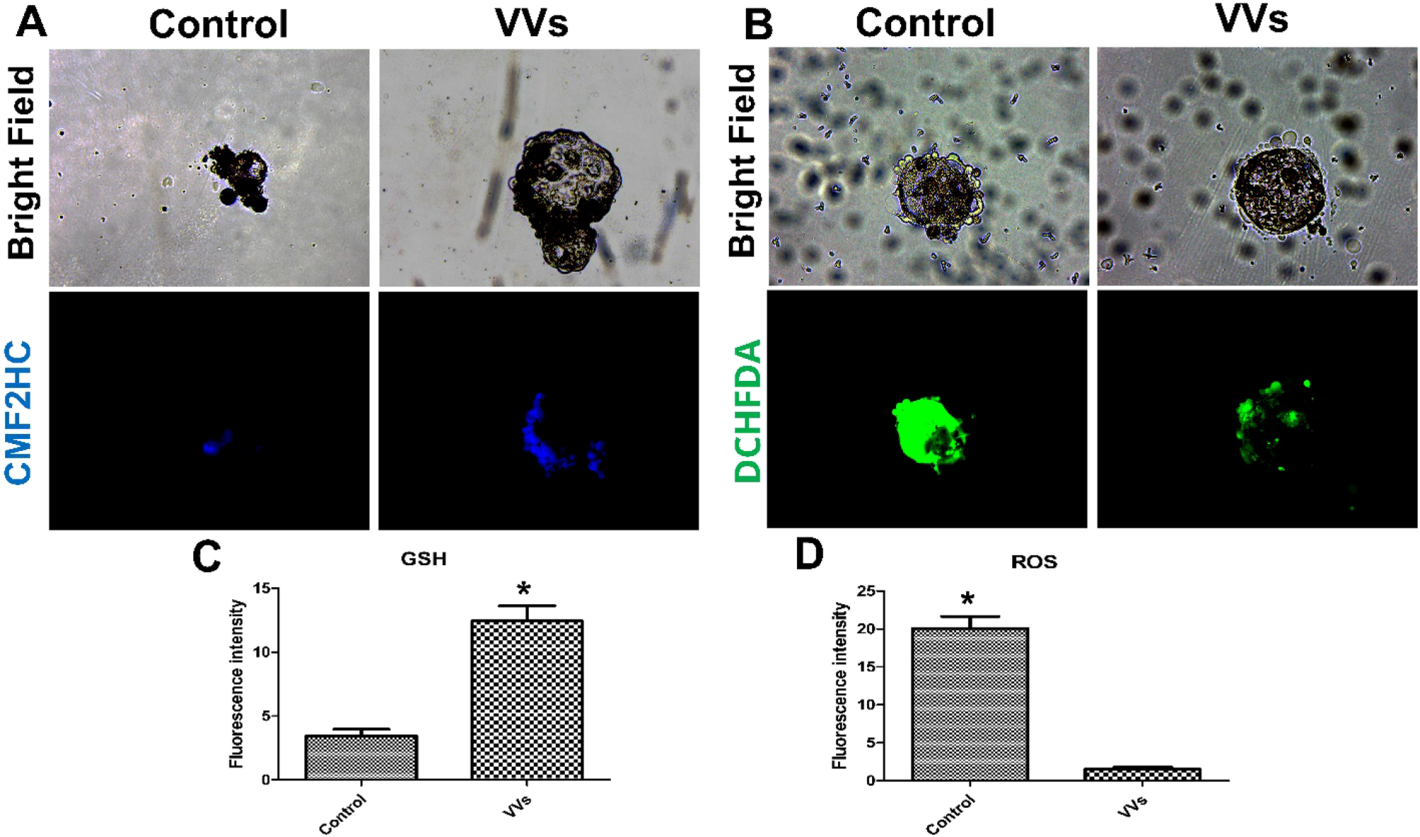
Effect of VVs on antioxidative status of embryos. (**A**) Reduced glutathione (GSH) was detected with CMF2HC stain and expressed as blue fluorescence, while reactive oxygen species (ROS) were stained with H2DCFDA and expressed as green fluorescence (**B**). (**C** and **D**) graphs show the comparison between the fluorescence intensity and values (mean ± SD) compared with Student’s t-test. Values denoted by an asterisk (*) were considered statistically different (*p* < 0.05).

**Figure 8:**
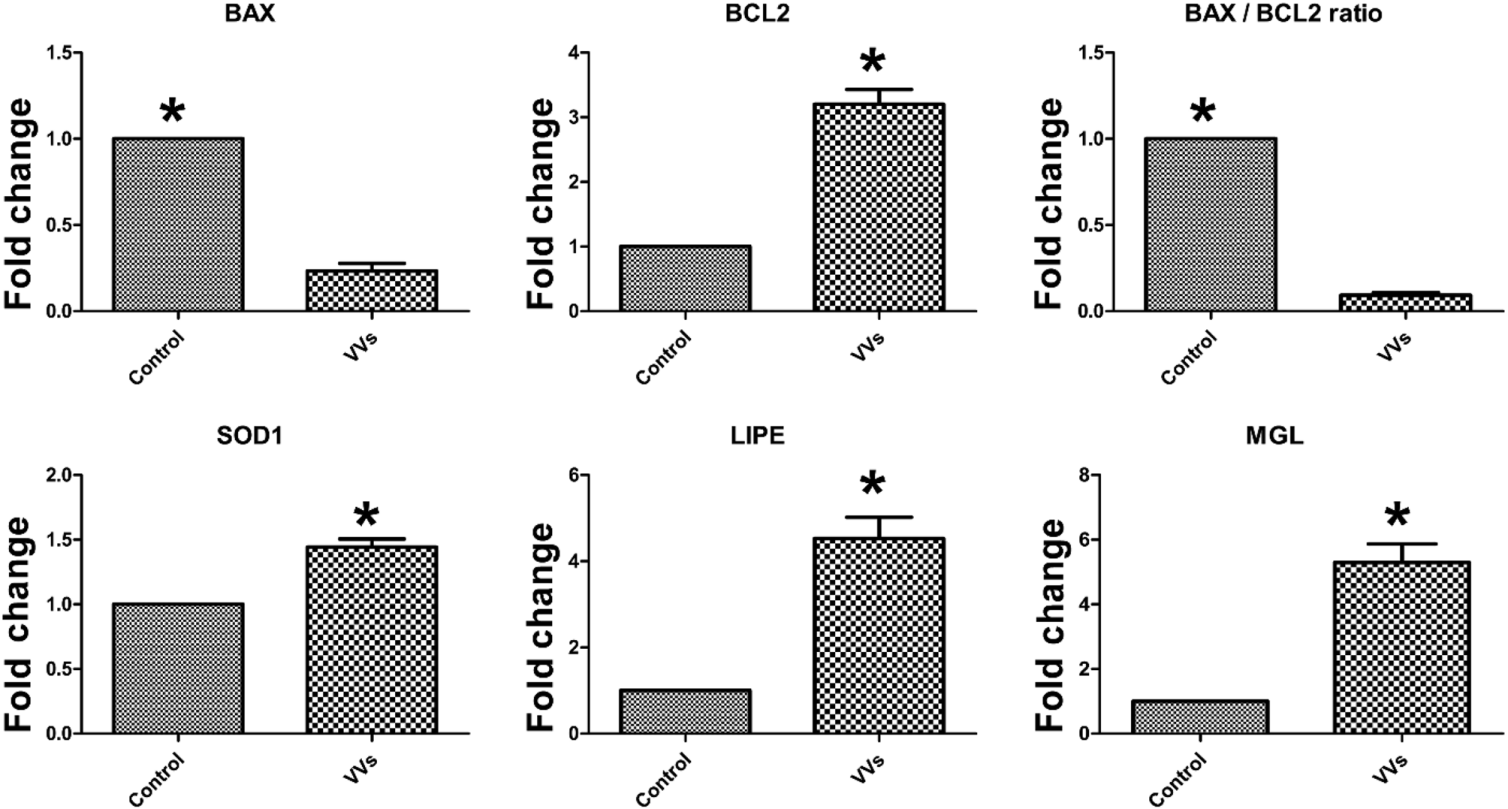
Relative quantitative analysis (RT-qPCR) of mRNA transcripts expressed in the embryos treated with VVs. Five blastocysts from each group of four replicates were used for qPCR analysis. The means were normalized to the control group and expressed as arbitrary units (fold change). Data are expressed as mean ± SD and the difference between the two groups was compared using Student’s t-test. Values denoted by an asterisk (*) are considered statistically significant (*p* < 0.05). House-keeping genes ACTB and U6 were used for quantification of the target transcripts through the ΔΔCt method.

Supplementation of VVs with the embryo culture medium significantly increased GSH contents and reduced ROS generation (Fig. 7). Furthermore, the expressions of BCL2, SOD1, LIPE, and MGL in the embryos supplemented with VVs were significantly increased (Fig. 8). Conversely, the expression of BAX and BAX/BCL2 ratio were significantly reduced with the supplementation of VVs.

## Discussion

For the first time, we successfully isolated and characterized the protein and miRNA contents of bilipid-layer nanovesicles of the chicken egg yolk “vitellovesicles (VVs)” and revealed their embryotrophic effects on porcine embryos. VVs contain exosome and microvesicle markers such as CD81, prominin, annexin A6, cystatin C, albumin, and Hsp70^33–36^. VVs contain several proteins and mature miRNAs that are essential for supporting early embryonic development.

Albumin from human and bovine sources has been used extensively for manufacturing embryo culture media in different animal species as a source of protein^37–39^. In addition, some other proteins were found and possess some embryotrophic effects and facilitate the immune and metabolic activities of the embryos such as immunoglobulin, apolipoprotein B, and IGF-binding proteins^40^. Moreover, VVs contain bone morphogenetic protein 2, retinol-binding protein, and exosome cargo such as fibronectin and CD81 that can support embryonic development^41–44^.

Some contents of VVs were linked to lipid metabolism such as apovitellenin (a major very-low-density lipoprotein component)^45^ and apolipoproteins^46^. Pre-implantation mammalian embryos were proved to actively utilize, synthesize, and store lipids as lipid droplets^47^ and therefore, activation of lipases will trigger the mobilization of accumulated lipids in response to different metabolic requirements^48,49^. Our results indicated an increase in MGLL and LIPE, hormone-sensitive type mRNA expression that subsequently promote lipid metabolism and enhance cell proliferation, survival, and developmental competence of embryos^16,50–53^.

The redox defense is also expressed in the cargo contents of VVs. Thioredoxin is a protein disulfide oxidoreductase that transfers two electrons and two protons to oxidize two cysteine thiol groups to a disulfide and a dithiol^54^. Moreover, the presence of ovotransferrin, which possesses antioxidant activity in addition to its role in iron metabolism^55^, explains the improved oxidative defense (increased GSH and SOD expression) and reduced ROS in the current results. Additionally, glutathione peroxidase 3 was also found in VVs as a major player in hydrogen peroxide catabolic process and oxidative stress defense^56^. Moreover, the increase in the antiapoptotic BCL2 mRNA expression and decrease in the apoptotic Bax expression and Bax/Bcl2 ratio explain the reduced apoptosis in the embryos supplemented by VVs. Taken together, the proteomics data indicate the involvement of the VVs protein in inhibiting peptidases, and endopeptidases, and the regulation of actin cytoskeleton which all protects the cells against the apoptosis induction^57–59^.

Tetraspanins and transgelin were also expressed in the cargo of VVs and this protein is a TGFβ signaling pathway that is essential in stem cell differentiation^60^ and importantly required for trophoblast implantation by promoting actin polymerization^61^, which can interpret the improvement in embryo attachment after supplementation of VVs.

The mature miRNAs present in VVs are of interest because we were able to define 21 of them as potential target mRNAs, which are essential for embryo development, cell metabolism, and differentiation of stem cells (References 64–87 in Table 3). Interestingly, unlike results of Wade et al.^5^ who detected only four miRNAs such as gga-miR-30c-5p, gga-miR-99a-5p, gga-miR-92-3p, and gga-miR-100-5p, we found 10 miRNAs expressed in VVs such gga-miR-92-3p, gga-miR-22-5p, gga-miR-26a-5p, gga-miR-100-5p, gga-miR-10a-5p, gga-miR-99a-5p, gga-miR-30c-5p, gga-let-7f.-5p, gga-miR-10b-5p, and gga-miR-204 (Table 3). Although, we found some miRNAs that are commonly expressed in the profile of the analyzed samples (Fig. 3), there is still some variability that might be due to the individual variations^5^ or the sequencing method^62^.

Interestingly, the ultrastructure of egg yolk LDL and HDL micelles were recently evaluated through TEM, cryogenic TEM, and the phosphorus-31 nuclear magnetic resonance and yielded small rounded or irregular particles of 17 to 200 nm in size^63–67^.

## Conclusion

We provide a detailed method for the isolation and molecular cargo contents of proteins and miRNA in addition to the functional characterization of egg yolk vesicles and their embryotrophic effects on the porcine embryo model. These VVs can be used for the formulation of an in vitro*–*culture medium for supporting different mammalian embryos development and could be engineered and used as a vehicle for nucleic acids for therapeutic purposes.

## Supplementary Information

Supplementary Information 1.

Supplementary Information 2.

Supplementary Information 3.

Supplementary Information 4.

Supplementary Information 5.

Supplementary Information 6.

Supplementary Information 7.

Supplementary Information 8.

Supplementary Information 9.

Supplementary Information 10.

Supplementary Information 11.

Supplementary Information 12.

Supplementary Information 13.

## Data Availability

The data supporting the findings of this study are included in the manuscript. Proteomics data generated during the current study are available in ProteomeXchange dataset PXD038679 [doi:@10.25345/C5RV0D524]. RNA sequencing dataset generated during the current study is available in the NCBI GEO (https://www.ncbi.nlm.nih.gov/geo/query/acc.cgi?acc=GSE219218).

## List of abbreviations

COC: Cumulus-oocyte complex
GSH: Reduced glutathione
HEPES: N-2-hydroxyethylpiperazine-N′-ethane-sulphonic acid
IVM: In vitro maturation
miRNA: Micro-RNA
mRNA: Messenger RNA
NTA: Nanoparticles tracking analysis
PBS: Phosphate-buffered saline
PCR: Polymerase chain reaction
PPI: Protein–protein interaction
PVA: Polyvinyl alcohol
RNA-seq: RNA sequencing
ROS: Reactive oxygen species
sRNAs: Small RNAs
ssCir DNA: Single-stranded circle DNAs
TEM: Transmission electron microscopy
TUNEL: TdT-mediated deoxyuridine triphosphate (dUTP-X) nick end labeling
VVs: Vitellovesicles
