# Supplementary material for: Isolation, characterization, proteome, miRNAome, and the embryotrophic effects of chicken egg yolk nanovesicles (vitellovesicles)
Source: Sci Rep. 2023 Mar 14;13:4204. doi: 10.1038/s41598-023-31012-0 (PMC10014936; doi:10.1038/s41598-023-31012-0)
Supplement: Supplementary file 2 — Supplementary Information 2. [file 41598_2023_31012_MOESM2_ESM.docx]

**Supplementary Table S2:** Sequencing results (clean or filtered) data statistics in the three different samples of VVs.

| Sample Name | Raw tag count | Clean tag count | Average Read Length | Q20(%) |
| --- | --- | --- | --- | --- |
| VVs-1 | 20,800,000 | 14,166,545 | 20.78 | 98.63 |
| VVs-2 | 33,760,000 | 14,123,933 | 17.93 | 98.59 |
| VVs-3 | 22,400,000 | 14,125,238 | 19.19 | 98.56 |
