# Supplementary material for: Isolation, characterization, proteome, miRNAome, and the embryotrophic effects of chicken egg yolk nanovesicles (vitellovesicles)
Source: Sci Rep. 2023 Mar 14;13:4204. doi: 10.1038/s41598-023-31012-0 (PMC10014936; doi:10.1038/s41598-023-31012-0)
Supplement: Supplementary file 3 — Supplementary Information 3. [file 41598_2023_31012_MOESM3_ESM.docx]

**Supplementary Table S3**: Results of mapping small RNAs (miRDeep2.pl*) of the three different samples of VVs.

|  | **All reads** | | | | | **Filtered reads (>17 nucleotides)** | | | | | **miRNA-annotated** | | | | | |
| --- | --- | --- | --- | --- | --- | --- | --- | --- | --- | --- | --- | --- | --- | --- | --- | --- |
| Sample | Reads | Mapped | Unmapped | Mapped % | Unmapped % | Reads | Mapped | Unmapped | Mapped % | Unmapped % | Reads | Mapped | Unmapped | Mapped % | Unmapped % |  |
| VVs-1 | 14,166,545 | 1,037,063 | 13,129,482 | 7.3% | 92.7% | 7,767,396 | 336,028 | 7,431,368 | 4.3% | 95.7% | 7,766,441 | 16,197 | 7,750,244 | 0.2% | 99.8% |  |
| VVs-2 | 14,123,933 | 1,215,059 | 12,908,874 | 8.6% | 91.4% | 5,445,316 | 113,622 | 5,331,694 | 2.1% | 97.9% | 5,444,945 | 1,835 | 5,443,110 | 0.0% | 100.0% |  |
| VVs-3 | 14,125,238 | 1,134,267 | 12,990,971 | 8.0% | 92.0% | 6,292,420 | 239,204 | 6,053,216 | 3.8% | 96.2% | 6,291,694 | 13,405 | 6,278,289 | 0.2% | 99.8% |  |

* analysis by the software miRDeep2.0.0.8 (<https://github.com/Drmirdeep/miRDeep2>)
