# Supplementary material for: Isolation, characterization, proteome, miRNAome, and the embryotrophic effects of chicken egg yolk nanovesicles (vitellovesicles)
Source: Sci Rep. 2023 Mar 14;13:4204. doi: 10.1038/s41598-023-31012-0 (PMC10014936; doi:10.1038/s41598-023-31012-0)
Supplement: Supplementary file 8 — Supplementary Information 8. [file 41598_2023_31012_MOESM8_ESM.docx]

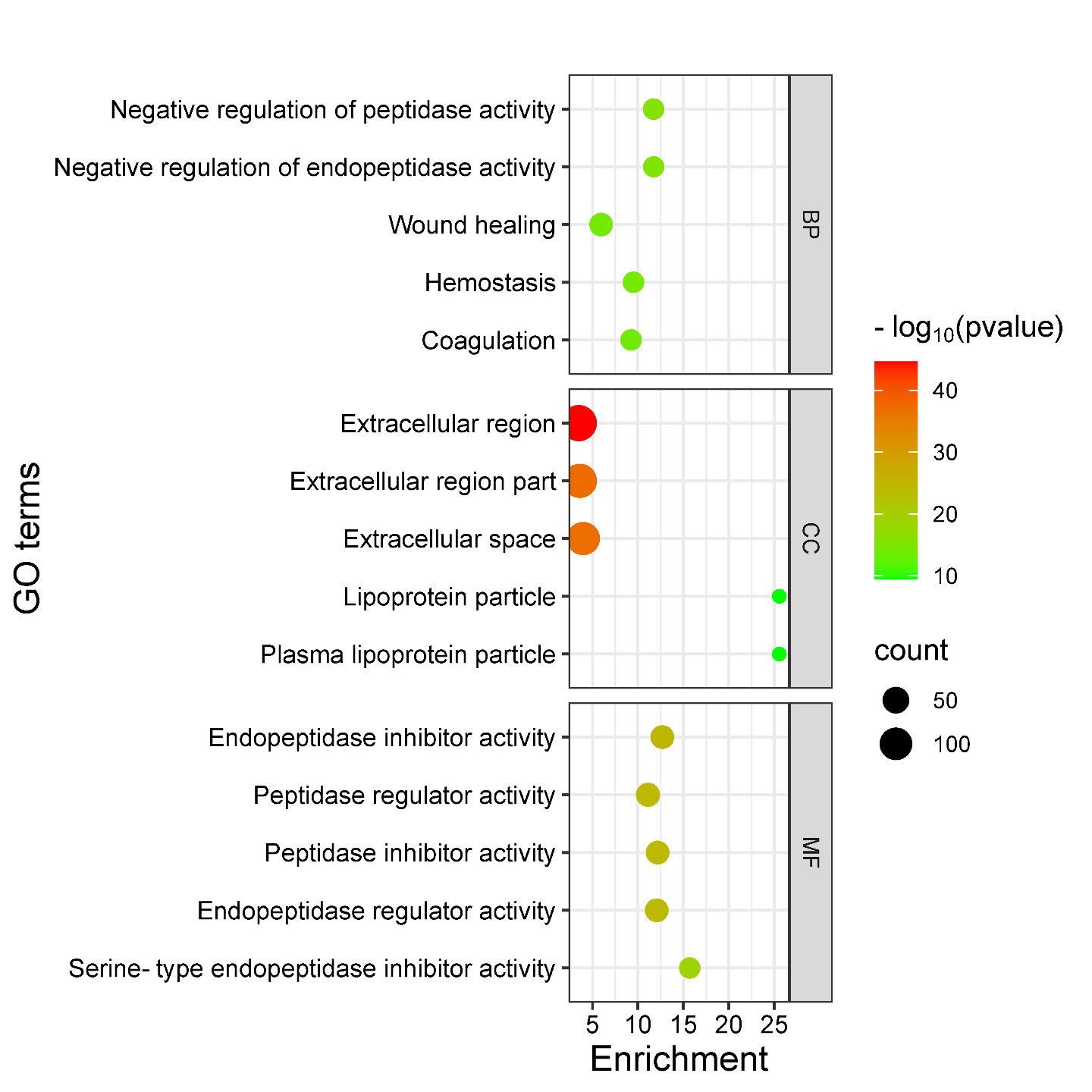


Supplementary Figure 1 (SF1): Bubble plot of VVs proteins showing the GO-BP, CC, and MF. p-value indicates the significance of the GO terms.
