# Supplementary material for: Isolation, characterization, proteome, miRNAome, and the embryotrophic effects of chicken egg yolk nanovesicles (vitellovesicles)
Source: Sci Rep. 2023 Mar 14;13:4204. doi: 10.1038/s41598-023-31012-0 (PMC10014936; doi:10.1038/s41598-023-31012-0)
Supplement: Supplementary file 10 — Supplementary Information 10. [file 41598_2023_31012_MOESM10_ESM.docx]

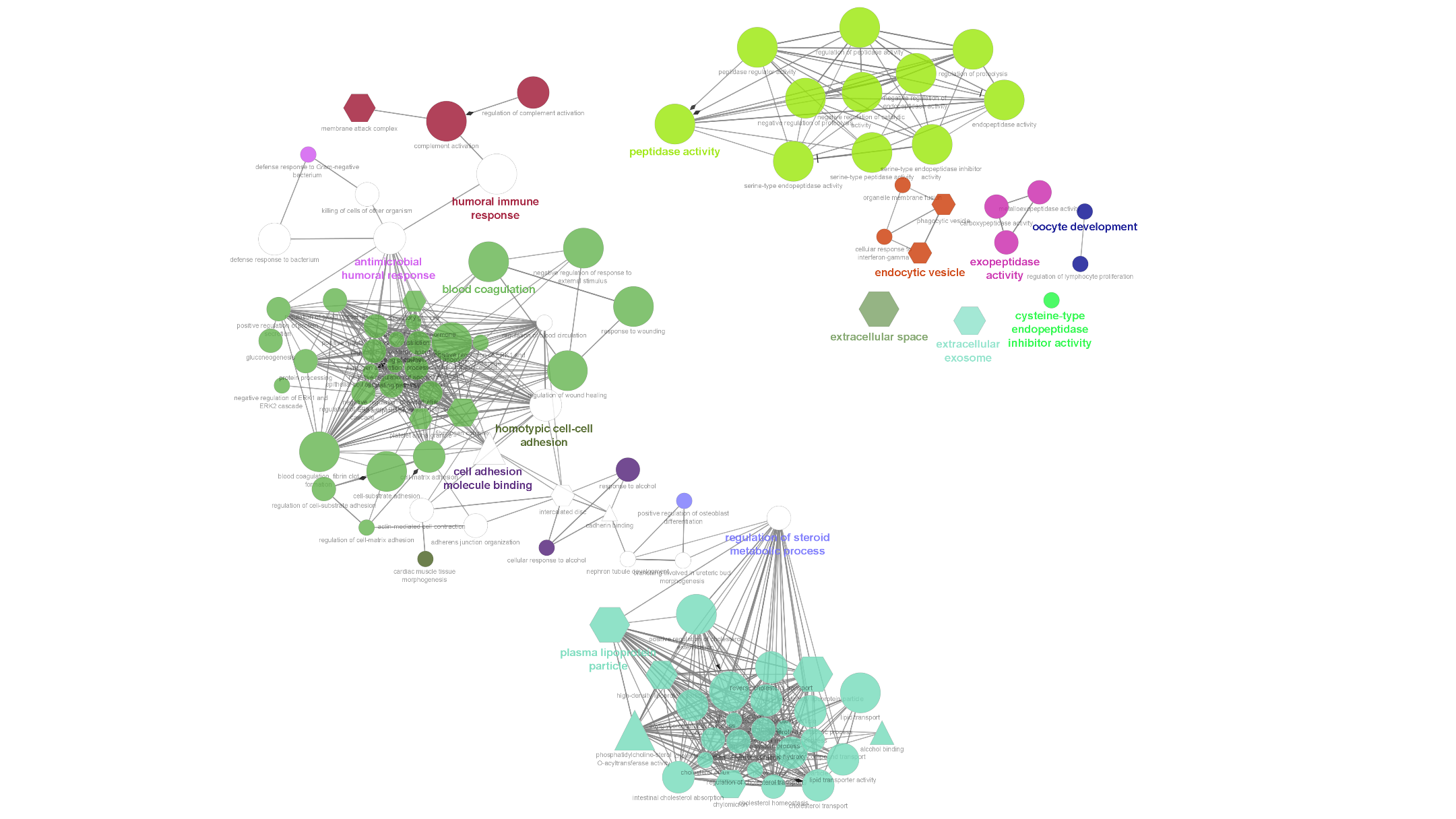


Supplementary Figure 3 (SF3): protein-protein interaction network (PPI) including gene ontology biological process (GO-BP), gene ontology cellular component (GO-CC), and gene ontology molecular function (GO-MF).
