# Supplementary material for: Isolation, characterization, proteome, miRNAome, and the embryotrophic effects of chicken egg yolk nanovesicles (vitellovesicles)
Source: Sci Rep. 2023 Mar 14;13:4204. doi: 10.1038/s41598-023-31012-0 (PMC10014936; doi:10.1038/s41598-023-31012-0)
Supplement: Supplementary file 11 — Supplementary Information 11. [file 41598_2023_31012_MOESM11_ESM.docx]

**Supplementary Figure 4 (SF4)**: Results of mapping and predicting small RNAs (miRge2.0*) in the egg yolk VVs samples (E1-E3).


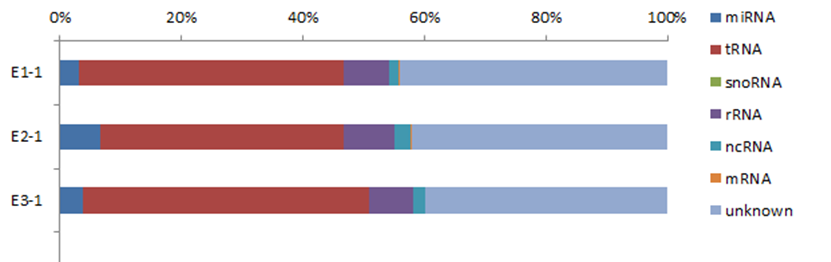


* Analysis performed through miRge2.0 software (<https://github.com/luketerry/miRge-2.0>).
